# Environmental and Genetic Determinants of Ankylosing Spondylitis

**DOI:** 10.3390/ijms25147814

**Published:** 2024-07-17

**Authors:** Rafał Bilski, Piotr Kamiński, Daria Kupczyk, Sławomir Jeka, Jędrzej Baszyński, Halina Tkaczenko, Natalia Kurhaluk

**Affiliations:** 1 Department of Medical Biology and Biochemistry, Collegium Medicum in Bydgoszcz, Nicholaus Copernicus University, M. Karłowicz St. 24, 85-092 Bydgoszcz, Poland; 2 Department of Medical Biology and Biochemistry, Division of Ecology and Environmental Protection, Collegium Medicum in Bydgoszcz, Nicolaus Copernicus University in Toruń, M. Skłodowska-Curie St. 9, 85-094 Bydgoszcz, Poland; 3 Department of Biotechnology, Institute of Biological Sciences, Faculty of Biological Sciences, University of Zielona Góra, Prof. Z. Szafran St. 1, 65-516 Zielona Góra, Poland; 4 Department of Rheumatology and Connective Tissue Diseases, Collegium Medicum, Nicolaus Copernicus University, University Hospital No. 2, Ujejski St. 75, 85-168 Bydgoszcz, Poland; 5 Institute of Biology, Pomeranian University in Słupsk, Arciszewski St. 22 B, 76-200 Słupsk, Poland

**Keywords:** ankylosing spondylitis, oxidative stress, pathophysiological state, reactive oxygen species, HLA, IL-23, IL-17

## Abstract

Exposure to heavy metals and lifestyle factors like smoking contribute to the production of free oxygen radicals. This fact, combined with a lowered total antioxidant status, can induce even more damage in the development of ankylosing spondylitis (AS). Despite the fact that some researchers are looking for more genetic factors underlying AS, most studies focus on polymorphisms within the genes encoding the human leukocyte antigen (HLA) system. The biggest challenge is finding the effective treatment of the disease. Genetic factors and the influence of oxidative stress, mineral metabolism disorders, microbiota, and tobacco smoking seem to be of great importance for the development of AS. The data contained in this review constitute valuable information and encourage the initiation and development of research in this area, showing connections between inflammatory disorders leading to the pathogenesis of AS and selected environmental and genetic factors.

## 1. Introduction

Ankylosing spondylitis (AS) is a chronic disease of an unknown etiology, belonging to the group of spondyloarthropathy together with rheumatoid arthritis (RA) and psoriatic arthritis (PsA) [1,2]. The reasons for its occurrence are believed to be genetic and environmental factors [3,4]. Due to its autoimmune nature, AS is also difficult to diagnose before pain symptoms occur. Researchers have proven the presence of genetic links between people affected by the described disease [4,5]. However, modern works indicate an interaction of genetic and environmental factors in the pathogenesis of AS [3,4]. The essence of the disease is inflammatory changes in the synovium of the sacroiliac joints, consisting mainly of the infiltration of lymphocytes and plasma cells. Over time, the spaces in these joints slowly heal and ossify. As the disease progresses, it leads to similar changes in the apophysial joints of the spine. The next stage of changes is inflammatory ossifications in the perivertebral tissue and in the outer layer of the fibrous ring. Vertebrae that are adjacent to each other are connected by syndesmophytes (bone bridges) [6,7,8].

**Figure 1:**
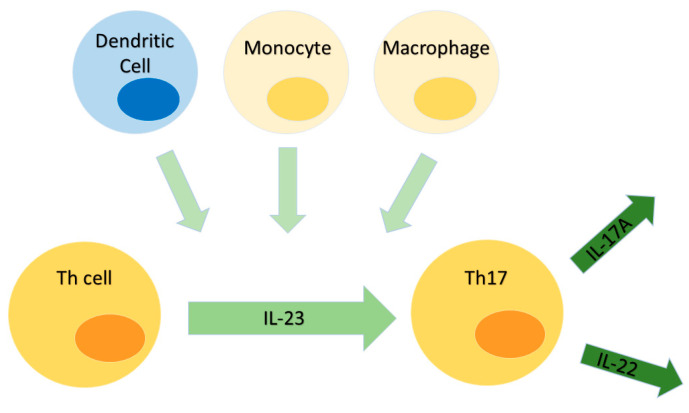
The schematic image of the IL-23/IL-17 pathway.

The pathogenesis of AS is not fully explained and the mechanisms underlying it are influenced by many factors. However, their basis is the inflammatory process resulting from the autoimmune response. The role of the IL-23/Il-17 pathway in autoimmune diseases has been proven by many researchers [9,10]. IL-23 is a heterodimeric cytokine secreted by monocytes, macrophages, and dendritic cells, often in response to information on threats [Figure 1]. IL-23 is crucial for the differentiation of helper T cells to the Th17 phenotype, as it polarizes and stabilizes this pro-inflammatory phenotype [11]. IL-17A is a pro-inflammatory cytokine also involved in maintaining mucosal immunity, especially in the skin and intestines. It is produced by various cell types, including CD4 cells (TH17), CD8+ T cells, and innate lymphoid cells [12]. The Il-23/Il-17 pathway is important in AS, as well as inflammatory bowel disease [13]. The concentration of IL-23 and IL-17 cytokines in these diseases is significantly increased, and it has also been shown that AS can be significantly alleviated by blocking the IL-23/IL-17 pathway, which further indicates the involvement of the discussed pathway in the pathogenesis of AS [14]. Pathogen-associated molecular patterns (PAMPs) activate TNF-α through the NF-κB pathway, which contributes to the expression of IL-17 [15]. Additionally, intracellular Nod-like receptors (NLRs) are able to recognize PAMPs. NLRs can then cause pro-inflammatory cytokines, such as IL-18, to be activated [16]. Modern scientists are searching for evidence of NLRP3 (NLR Family Pyrin Domain Containing 3), YAP (Yes1 Associated Transcriptional Regulator), and AMPK (AMP-activated protein kinase) pathway participation in the pathogenesis of AS. There is evidence that NLRP3 plays a significant role in human immunity and autoimmune disease development. Research conducted on mice proved the influence of the AMPK/YAP/NLRP3 signaling pathway in the pathogenesis of AS [16,17,18].

The hypothesis of the activation of bone morphogenetic protein signals and the reduction in sclerostin and Dickkopf-related protein 1 (DKK1), which are endogenous Wnt pathway inhibitors, is somewhat supported by the available data [19,20,21]. According to the modified Stoke Ankylosing Spondylitis Spine Score, radiographic damage may be correlated over time with disease activity [22].

Angiogenesis is an important stage in the development of rheumatic diseases. Patients with AS also have an increased atherosclerosis risk, therefore leading to an increased mortality from cardiovascular diseases [23]. Vascular endothelial growth factor (VEGF) is a key regulator of angiogenesis, with several isoforms and roles in different physiological processes. Mounting data indicate a correlation between the VEGF system and rheumatic diseases with anti-VEGF and VEGF receptor (VEGFR) [24]. Angiogenesis is a hypothesis that explains the process of new bone creation in AS. New bone growth, sacroiliitis, and enthesitis, which are different symptoms of AS, necessitate angiogenesis. VEGF plays a key role in regulating this process, according to Yamamoto’s research in 2013 [25]. Fearon et al. noted that VEGF is present in early inflammatory arthritis and has a strong connection to angiopoietins [26]. It may play a role in regulating these processes in the development of early AS. VEGF can directly control the development of osteoblasts from synovial fibroblasts, which aids in the production of new bone in AS [27]. Furthermore, according to Kawashima’s research in 2009, it can activate the COX2 pathway [28]. According to Drouart, VEGF is also involved in inflammation in AS [29]. Multiple investigations have confirmed that VEGF is associated with AS susceptibility, indicating that it is involved in the development of AS [30].

In this review article, we are trying to find the connection between the inflammatory processes underlying the pathogenesis of AS with selected genetic and environmental factors such as oxidative stress, the concentrations of selected elements, lifestyle, gut microbiota, infections, and genetic polymorphisms. Although the etiology of this disease is not fully known, environmental and lifestyle factors, which are modifiable factors, are implicated. Analysis of the environment in the form of pollutants and toxic substances can provide information about changes occurring at the cellular level, helping to better understand the pathomechanism of AS.

## 2. The Participation of Oxidative Stress in Pathogenesis of AS

Oxidative stress refers to an imbalance that favors oxidants over antioxidants, which can cause molecular damage and/or the disturbance of redox signaling and control [31]. More specifically, oxidative stress is defined as a disturbance in the balance between the production of reactive oxygen species (free radicals and non-free radicals) and antioxidant defenses [32]. Researchers’ reports suggest that, in the chronic inflammatory process, significant DNA damage is involved. Oxidative stress is a phenomenon harmful to DNA, as evidenced by its numerous damages in the course of action of reactive forms of oxygen [33]. The production of inflammatory cytokines in the course of disease development will induce the production of oxidative stress markers [34]. Therefore, despite the fact that the etiology of AS is still not fully known, we cannot ignore the significant share of oxidative stress underlying AS, which has been proven by researchers [35].

The stimulation of the cell membrane during phagocytosis activates NADP oxidase, which catalyzes the formation of the superoxide anion O^2−^. Thanks to the presence of superoxide dismutase (SOD), this radical is converted into hydrogen peroxide and singlet oxygen [36,37]. The reaction between the superoxide radical and H_2_O_2_ produces a hydroxyl radical. These three compounds are called reactive oxygen species (ROS) and play a significant role in inflammatory processes [36]. In the context of changes in the joints, the hydroxyl radical has the greatest share, having the ability to combine with glycoproteins, phospholipids of cell membranes, hyaluronic acid, DNA, and collagen, leading to the destruction of their structure [38,39]. The body has a number of defense mechanisms aimed at neutralizing the risk of changes caused by free oxygen radicals. The body’s natural antioxidant barrier consists of a number of chemical compounds that help to reduce and inactivate ROS. Antioxidant defense mechanisms can be divided into enzymatic and non-enzymatic. The first group includes enzymes that catalyze ROS scavenging reactions—superoxide dismutase, catalase CAT, peroxidase (GPx), Glutathione S-transferase (GST), and glutathione reductase (GR) [36,37]. The second group includes compounds obtained from external sources, such as vitamins C, E, flavonoids, and β-carotene, but also those synthesized by the body, such as melatonin and glutathione [40]. Oxidative stress can trigger a large number of proteins such as heat shock proteins and turn on transcription factors such as AP-1 and NF-κB [41,42,43].

In inflammatory diseases, there is an increased production of superoxide anions at the site of inflammation [44]. This problem is important for the pathomechanism of many diseases, including AS discussed in this paper, RA, and PsA [37,45,46,47,48].

An important mechanism of action of oxidative stress is the disruption of redox signaling, causing molecular damage that inevitably affects angiogenesis, inflammation, and the function/activation of dendritic cells, lymphocytes, and keratinocytes [49]. This is also important for cytochrome C, which floats in the peripheral mitochondrial membrane and mediates the transfer of electrons in the respiratory chain [50,51,52]. The formation of reactive oxygen species induces the release of cytochrome c into the cytosol [53], where it binds to the apoptosis protease activation factor-1 (APAf-1), forming an apoptosome from ATP or dATP; this complex activates procaspase 9, which triggers an enzymatic cascade leading to cell apoptosis [54,55,56]. In this way, oxidative stress causes uncontrolled cell apoptosis, among others within the joint tissue, leading to its damage and inflammation [57]. Several clinical studies on diseases characterized by cell death, either due to apoptosis or necrosis, have reported the release of cytochrome c from the mitochondria into the extracellular space and ultimately into the circulation. Elevated levels of cytochrome c in serum have been found in chronic and acute diseases, including arthritis, myocardial infarction and stroke, and liver diseases [58,59,60]. Its role as an inducer of skin and joint inflammation has been proposed. In particular, an association between cytochrome c, skin inflammation, and keratinocyte proliferation during ROS production has been demonstrated [61]. The proliferation of keratinocytes is the basis of the pathomechanism of diseases such as psoriasis and PsA [62,63]. Dysregulations in ROS and cytochrome c release have been associated with psoriasis and been reported to cause associated skin inflammation [64]. This is one of the mechanisms that facilitates their growth and, at the joint level, the destruction of articular cartilage and the erosion and/or proliferation of bones [65]. Based on the important role played by ROS in some malignancies and rheumatic diseases, it is also possible that cells are involved in the activation of AS [66]. In patients with AS with inflammatory bone disease located in the lower part of the spine, an increase in the total oxidative status, as well as the advanced oxidation of protein products and lipid peroxidation, was demonstrated [35,45,67,68].

A significant role in the development of this disease is believed to be attributed to the disturbance of the body’s oxidative balance [45]. Over recent years, the importance of disturbing the balance between the production of ROS and the ability to inactivate them via the antioxidant system in disturbances of body homeostasis has been increasingly discussed. There are studies that have confirmed the significant role of oxidative stress in the pathogenesis of chronic inflammatory autoimmune diseases [69]. AS may reduce the body’s condition because it disturbs the balance between the interactions of individual antioxidant enzymes. This is related to changes in their activity compared to healthy people in whom no pathological changes in the osteoarticular system are observed [35]. Moreover, excess free radicals may contribute to the damage of biological structures due to the oxidation processes of the compounds present in them [70]. Free oxygen radicals cause the oxidation of proteins, lipids, and DNA, and, thus, may contribute to tissue damage. ROS sources can be divided into endogenous and exogenous. Endogenous sources include numerous biochemical processes taking place in physiological conditions, and the most efficient source is the respiratory chain. In turn, external factors include, among others, environmental pollution, tobacco smoke, ionizing radiation, ultraviolet radiation, and ultrasound [71]. As a result of the oxidation reaction, toxic products are formed, which, by their action on the cell, have a cytostatic effect and lead to damage to cell membranes and the activation of apoptosis mechanisms [72]. The body has a natural defense system that protects cells against excessive amounts and harmful effects of ROS. The pro- and antioxidant balance is ensured by antioxidant enzymes, including SOD, CAT, GPx, and GR and a number of non-enzymatic substances, including glutathione, vitamin E or vitamin C, and ceruloplasmin (CP). These are compounds that, through their action, enable the removal of excess reactive oxygen species from the cell. Small-molecule antioxidants are mostly compounds of exogenous origin that are supplied to the body through the diet. They act as responses to non-specific reactions that result in the inactivation of free radicals [73]. ROS can have a significant impact on the development and progression of inflammatory conditions in the osteoarticular system, including ankylosing spondylitis [74]. Moreover, the increased production of ROS, which intensifies joint inflammation, may be related to a low supply of fish in the diet of AS patients. This fact is then explained by the low content of antioxidant vitamins and omega 3 acids, which may have a protective effect and counteract the effects of oxidative stress. As a result of the ongoing inflammatory process and excess ROS, the body’s oxidative reserves are depleted, and damage caused by ROS accumulates in the joints [75]. Stanek et al. [76] showed the reduced effectiveness of the natural antioxidant barrier in patients with AS. Karakoc et al. [45] described the total potential of antioxidant parameters in patients with AS compared to a group of healthy people. Their research also showed a reduced antioxidant potential in patients with AS. In turn, Ozgocmen et al. [67], in their study, did not show statistically significant differences in SOD activity between groups of AS patients and healthy people. However, in the study by Pishgahi et al. [77], oxidative stress parameters were assessed in AS patients with metabolic syndrome compared to AS patients without comorbidities. It turned out that, in patients with the above-mentioned syndrome, the levels of SOD and CAT were increased compared to patients with AS without concomitant diseases. In the study by Danaii et al. [78], a statistically significant variability was obtained regarding the parameters of oxidative stress in people with AS. The study involved 35 people with diagnosed disease and 40 healthy people who constituted the control group. Significantly increased levels of SOD and CAT were observed in the group of people suffering from AS. The total antioxidant status (TAS) was proven to be lower in patients with ankylosing spondylitis compared to healthy individuals [45,76,79].

An antioxidant that also belongs to the group of acute-phase proteins is ceruloplasmin. The concentration of this protein increases in chronic inflammation. Also, in the research conducted by Jayson et al. [80], significantly higher concentrations of ceruloplasmin were found in patients with AS. Similar results were shown in the study by Aiginger et al. [81], which found an increased level of ceruloplasmin among patients with AS compared to a group of healthy people. Similarly, in an experiment conducted by Li et al. [82], a higher ceruloplasmin expression was shown in AS patients compared to the control group. A high level of free oxygen radicals, combined with a deficiency in the activity of antioxidant enzymes, may result in damage to the body at the molecular level [72,75].

Research conducted by Salmón et al. [83] confirmed that antioxidant defense mechanisms adapt to greater amounts of pollution in the urban environment, but not enough to fully neutralize the damage caused by greater exposure to oxidative stress.

The oxidation reactions of unsaturated fatty acids proceed in stages and lead to the formation of end products harmful to the body, including malondialdehyde (MDA) as an indicator of lipid peroxidation and advanced oxidation protein products (AOPP) [84]. These are compounds that can inhibit the processes of replication, transcription, and DNA strand breaks. They can also inhibit the activity of selected enzymes or change the antigenic properties of proteins. MDA and AOPP, respectively, can function as secondary mediators to exacerbate damage and accelerate the course of the disease by stimulating the TNF-α and NF-κB pathways, which are critical for the inflammatory process associated with AS [85]. In the study by Kozaci et al. [34], a higher level of MDA was found among patients with AS compared to the control group. The results of Ozgocmen et al. [67] and Ayala et al. [86] are similar.

The oxidative biomarkers in the serum that were higher in AS patients and the part that oxidative stress plays in the pathophysiology of AS may be explained by the following theories. First off, oxidative stress can harm proteins, lipids, nucleic acids, cell membranes, and the extracellular matrix, which raises the concentration of oxidative injury products [87]. When leukocytes become more activated at the site of inflammation in AS, ROS and enzymes like Myeloperoxidase (MPO) are released, which can cause hypochlorous acid to form. By reacting with the components of cartilage, hypochlorous acid causes tissue damage and protein oxidation. [68,85]. Second, by reacting with cell membranes and triggering phospholipase A2, lipid peroxidation affects the fluidity and receptor functions of cell membranes. Thus, it is possible to induce T cells to release more interleukins, which will set off a vicious cycle by increasing lipid peroxidation and inflammation [88]. The research conducted by Feng et al. showed promising results in mice by applying Punicalagin, which effectively decreased oxidative stress and regulated the NF-kβ pathway [89]. Kiranatlioglu-Firat et al. proved, in their research, the participation of oxidative stress, oxidative DNA damage, and chromosomal DNA damage in patients with AS [33]. Exogenous ROS stimulate the induction of VEGF expression in various cell types, such as endothelial cells, smooth muscle cells, and macrophages, where VEGF induces endothelial cell migration and proliferation through an increase in intracellular ROS [90,91,92,93]. Thus, the reciprocity between oxidative stress and angiogenesis is centered on the VEGF signaling pathway. A number of studies have demonstrated this positive interrelationship between ROS and angiogenesis. Hydrogen peroxide induces VEGF expression in vascular smooth muscle cells, as well as endothelial cells, thereby promoting angiogenic responses [90,92]. Oxidative stress enhances metabolism toward glycolysis, possibly leading to increased inflammatory processes and impaired angiogenesis in RA [94,95].

The overproduction of free radicals and weakening of mechanisms for their neutralization are two of the causes of the development of the inflammatory process in ankylosing spondylitis. The toxic accumulation of metabolites first leads to functional impairment and then the destruction of the structure of the cell membrane in cells, resulting in inflammation. As a result, a number of inflammatory mediators are secreted within the tissue. This process leads to uncontrolled apoptosis and tissue damage. In the pathogenesis of AS, a significant role is attributed to disorders in the pro- and antioxidant systems. Malfunctioning defense systems do not protect the body against the accumulation of free radicals; therefore, they are unable to protect against the development of inflammation and the destruction of individual structures. From this point of view, it seems important, among others, to supply exogenous antioxidants in the diet or avoid stimulants.

In recent years, the importance of disturbing the balance between the production of ROS and the ability of the antioxidant system to neutralize them has been increasingly discussed. The significant role of ROS in the pathogenesis of chronic autoimmune diseases has been confirmed. An excess of ROS can lead to damage to structures at the cell level due to the oxidation of the chemical compounds contained therein. Additionally, researchers wonder whether the increased synthesis of free radicals is a causative factor or whether it appears secondary to the disease. Therefore, conducting research on the involvement of ROS in the etiopathogenesis of AS is justified. The ongoing oxidation reactions may initiate a chronic inflammatory process and contribute to tissue destruction in the course of AS. From this point of view, antioxidant treatment seems to be beneficial, which may have a significant impact on the direction of AS pharmacotherapy. Moreover, the generation of significant amounts of free oxygen radicals is caused by an excessive influx of neutrophils, which leads to a disturbance of the pro- and antioxidant balance in cells and, consequently, to the uncontrolled and progressive destruction of structural elements of the joints [94,95,96]. Therefore, eliminating the phenomenon of oxidative stress in places affected by inflammation may significantly influence the inhibition of the development of the disease, improving the quality of life of patients. The levels of the antioxidant enzymes SOD, CAT, GPx, and GR were found to be elevated or reduced in certain research. One possible explanation is that the levels of antioxidant enzymes may not be reliable predictors of high or low oxidative stress. Instead, the evaluation of oxidative stress is mostly based on the harm it causes to important biological molecules such as proteins, lipids, and DNA. MDA serves as a marker for this assessment, as it is one of the byproducts of lipid peroxidation. Furthermore, TAS is an unreliable indicator of overall antioxidant status due to its use of manufactured free radicals as one of its reagents, which are not naturally produced in human bodies. Elevated levels of MDA and total oxidative stress (TOS) provide evidence of the influence of this phenomenon in individuals with AS. A summary of oxidative stress indicators is shown below (Table 1).

## 3. The Participation of Elements as Environmental Factors in AS Development

The importance of environmental factors in the etiopathogenesis of diseases of the osteoarticular system has been noted. The osteoarticular system plays a special role in assessing the risk of exposure to xenobiotics. Bone reconstruction and mineralization take a very long time, usually the entire life of the organism, so they may be a determinant of the long-term accumulation of bioelements and toxic metals. Unfavorable changes in the skeletal and joint systems are most often the result of the long-term impact of heavy metals on the body. It is suggested that elements easily absorbed by bone tissue, e.g., cadmium, lead, aluminum, and strontium, may be involved in the development of diseases of the osteoarticular system (osteoarthritis, rheumatoid arthritis, and osteoporosis). The proper functioning of the osteoarticular system is determined by various factors, depending on systemic and multi-organ changes [99,100,101]. Although bones or joint elements could be a very valuable material for assessing the state of elements in humans, except in special situations (orthopedic surgeries, e.g., arthroplasty), such material is difficult to obtain intravitally. Hence, most studies are usually based on material derived from serum. Metabolic diseases of the osteoarticular system are clinical syndromes developing as a result of the interaction of many factors, some of which are the impact of the external environment, including exposure to chemicals or elements that potentially act as triggers of autoimmune reactions [102]. Other environmental factors that play a significant role in the pathomechanism of AS include an abnormal element metabolism in the body and exposure to heavy metals and other pollutants [103].

**Figure 2:**
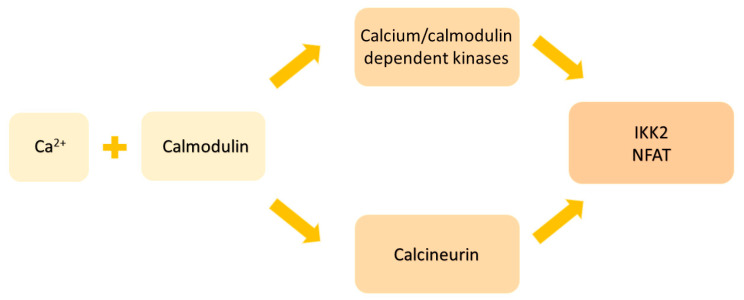
The schematic image of calcium activated immune response.

Calcium plays a role in inflammatory processes as a secondary messenger. Its abnormal deposition in the extracellular matrix, occurring during dystrophic or repair processes, is the basis for ectopic calcifications in bone and extraskeletal spaces. Ankylosing spondylitis is a rheumatic disease characterized by the inflammation and final calcification of tendon attachments, which are an important site for tendons, joint capsules, and ligaments [104]. Since AS is often accompanied by non-specific intestinal inflammation and disorders in its microflora, it is believed that it is important for calcium absorption. As a result of the above-mentioned comorbidities, the expression of Ca transporters such as calbindin D9K is inhibited, and, therefore, the absorption of calcium in the intestine is impaired [105]. Calcium can influence the immune response and repair mechanisms in several ways. Intracellular calcium can form complexes with calmodulin and activate calcium/calmodulin-dependent kinases or calcineurin, which, respectively, phosphorylate κB kinase inhibitor (IKK2) and dephosphorylated nuclear factor of activated T-cells (NFAT) [Figure 2].

This leads to the activation and translocation of either nuclear factor kappa light chain enhancer of activated B cells (NF-κB) or NFAT to the nucleus, where they can control the transcription of various genes related to proliferation and inflammation [106]. Additionally, the importance of calcium in inflammation has been revealed by the role played by calcium-sensing receptors (CaSR) in immune cells. In fact, hypocalcemia often occurs in sepsis or other systemic inflammatory conditions due to the inhibition of parathyroid hormone secretion. This may depend on the abnormal expression of calcium receptors on peripheral mononuclear cells, which use calcium to secrete cytokines and activate the inflammasome. Therefore, cytokines such as IL-6, TNF-α, and IL-1 may indirectly influence the secretion of parathyroid hormone (PTH) by increasing the expression and activation of calcium receptors on immune cells [107]. Additionally, intracellular calcium has been shown to be increased in neutrophils from AS patients, where it may contribute to lipid peroxidation, apoptosis, and the activation of caspases 3 and 9. Of note, the anti-TNF monoclonal antibody infliximab, used in the treatment of many rheumatic diseases, including AS, may prevent the influx of calcium into neutrophils, suggesting that the opening of calcium channels may be a result of the response to cytokines [108]. Similarly, a blockade of lymphocyte potassium channels may have anti-inflammatory properties in patients with AS and rheumatoid arthritis, preventing the differentiation of CD4+ and CD8+ T cells [109]. The calcium levels in the body can be influenced by vitamin D deficiency. In the case of this deficiency, calcium is reabsorbed from the bones to maintain the correct serum levels [110]. Chen et al., in their metanalysis, indicated a strong correlation between elevated levels of vitamin D in the blood and the ability to manage the disease and enhance the overall well-being of individuals with AS. Furthermore, the overall blood 25(OH)D levels and the inverse correlation between serum vitamin D levels and Bass Ankylosing Spondylitis Disease Activity Index in patients with AS may be considerably impacted by differences in continents and ethnicities. Thus, individuals diagnosed with AS are recommended to augment their vitamin D intake through either exposure to sunlight or the use of dietary supplements. This is enacted to reduce the severity of clinical symptoms and improve overall quality of life [111]. A metanalysis by Diao et al. showed similar results, proving that patients with higher levels of vitamin D had a lower C-reactive protein and erythrocyte sedimentation rate, although there were no significant changes in the serum calcium levels between patients with AS and healthy controls [112]. On the other hand, a two-sample randomized Mendelian study by Jiang et al. showed no strong evidence to connect vitamin D levels and AS. [113]. The vitamin D levels in the body are strongly connected to sun exposure and ethnicity. Although there is no evidence of differences in the serum calcium levels in patients with AS and healthy people, the real determinant of calcium–phosphate metabolism disorders would be an analysis of bone composition due to the involvement of factors such as vitamin D or parathromon. The level of vitamin D is significantly related to exposure to sunlight, which depends on the geographical location and ethnic origin of the patients.

Another important element that is a component of nervous tissue proteins is magnesium. The role of this element cannot be ignored due to its involvement in the bone mineralization process and its impact on the progression of AS [114]. It is responsible for the proper absorption of calcium in the bones, regulates the transport of this element, activates the ossification process, stimulates bone-forming cells to incorporate calcium into the bone structure, and also prevents pathological calcium deposition in soft tissues [115]. Moreover, by reducing the level of cytokines, i.e., factors that are crucial for pathological changes in the joints in the course of AS, it influences the body’s immune response. This element reduces the concentration of inflammatory factors, including TNF-α, interleukins IL-1β, IL-6, and NF-κB [116]. Tumor necrosis factor α stimulates NF-κB to bind to TNF receptors, leading to the activation of a kinase cascade. The above processes lead to the activation of pathological responses triggered in the course of AS [117]. Increased phosphorus concentrations lead to the release of calcium from the bones, thus increasing its content in the blood. This not only disturbs bone mineralization processes, but also leads to the induction of inflammatory reactions resulting from hypercalcemia. Researchers have shown reduced magnesium concentrations in the blood of people with AS [118].

Selenium, on the other hand, optimizes the action of glutathione peroxidase, one of the main enzymatic antioxidants responsible for the transformation of glutathione and combating reactive oxygen species. In case of its deficiency, the body’s antioxidant defense decreases, which may result in the development of a number of abnormalities. Its action is based on the metabolism of selenoglutathione. The two-step reaction of selenium reduction to hydride takes place in both the liver and erythrocytes. In the case of red blood cells, this is important because it protects hemoglobin against oxidation, which reduces its affinity for oxygen. Selenium takes part in the control of lipid peroxidation and nucleic acid synthesis processes. Thanks to its ability to penetrate cell membranes, it can induce the production of antibodies [119]. Selenoproteins containing selenocysteine have antioxidant activity, protecting the components of cell membranes and cytosol against reactive oxygen species. Selenium reduces the expression of inflammatory genes and may reduce the ability of matrix metalloproteinases (MMPs) to break down cartilage by inducing tissue inhibitors of MMPs (TIMPs). Selenoprotein P scavenges peroxynitrite, a potent inflammatory factor produced in inflamed joints. Selenium can inhibit neovascularization, which is crucial for the development and perpetuation of rheumatoid synovitis [119,120]. An incorrect concentration of selenium in the blood is an important factor for the osteoarticular system. Deficiency may lead to autoimmune changes in the joints in the course of diseases such as AS or RA [121]. Reduced selenium concentrations in the blood have been observed in patients with autoimmune diseases compared to healthy people. However, the supply of selenium in patients suffering from autoimmune diseases helps to alleviate their course [122]. The reasons for such improvement are believed to be the antioxidant properties of this element. In diseases such as RA, the administration of selenium helps to reduce ailments such as joint pain and swelling [123]. A recent meta-analysis [124] showed a significant difference in serum Se between RA patients and healthy controls. A meta-regression analysis determined that age, gender, and disease duration did not significantly affect serum selenium concentrations. However, such an effect has been demonstrated with the administration of steroids. Their use was positively associated with serum Se concentration. Significant differences in the Se concentration in the serum of RA patients on different continents were found. Hence, there is a suggestion that the relationship between RA and Se results from an insufficient intake of this element [124,125]. Another aspect is the influence of some factors on the bioavailability and activity of Se, such as geographical region, drugs, and genetic polymorphisms. Although clinical trials have been conducted to analyze the effect of Se on patients with RA, the expected results have not been obtained [126,127,128,129]. On the other hand, preclinical studies examining the effects of new selenium nanoparticles have yielded favorable results [130,131,132]. It seems necessary to conduct studies assessing both the concentration of Se in the diet and its concentration in the serum of this population in order to more comprehensively determine whether there is a relationship between the deficiency of this element and the progression of disease activity.

Zinc is one of the most important elements for the natural antioxidant barrier. It is necessary for the formation of superoxide dismutase. Zinc, as a cofactor in over 3000 human proteins and a signal ion, affects many pathways important for AW. It is known that this is not only a matter of zinc status, but also of mutations in many genes encoding proteins that maintain cellular zinc homeostasis, such as metallothioneins and zinc transporters from the ZIP (Zrt/Irt-like protein) and ZnT families. There are also many pathways that change the expression of these proteins [133]. Zinc also has regulatory influence on glutathione peroxidase and metallothionein [134,135]. A deficiency of this element can lead to a weakening of antioxidant defense and the induction of proinflammatory processes. In the case of arthritis, reduced Zn assimilation causes an increase in inflammatory status, immune system changes, extracellular matrix breakdown, Zn-mediated disruption of the Th1/Th2 balance with an increase in Th17, and a loss in PMN phagocytic activity. The mechanisms underlying this are related to an increased secretion of IL-1β, IL-8, and TNF-α and a reduced secretion of IFN-γ and IL-2 [136,137,138].

There are studies showing no connection between the concentrations of elements and the occurrence of AS. This study provides insufficient evidence to indicate a causal relationship between calcium, zinc, copper, and selenium and AS, however, the authors suggested that more extensive research should be conducted on this matter [139]. According to the literature data, an assessment of the zinc content in the hair of non-smokers and smokers with RA showed significantly reduced zinc concentrations in both groups [140]. Similarly, the total level of zinc in the serum of people with RA was low and negatively correlated with the levels of secreted pro-inflammatory markers [124,141]. A comprehensive review of the pathogenesis of RA from a medical point of view did not mention Zn at all [142]. As has been shown in many studies, zinc is involved in arthritis because it plays an essential role in the innate and adaptive parts of the immune system, in the regulation of various aspects of the inflammatory response, and in bone growth and regeneration [143,144,145]. The assessment of the zinc content in the hair of non-smokers and smokers with RA showed significantly reduced zinc concentrations in both groups [140]. A large meta-analysis of 16 studies showed a similar correlation for zinc and selenium and an inverse correlation for copper (increased Cu concentration vs. control group) [124].

Cadmium disturbs the functions of zinc, and zinc deficiency causes its increased absorption. Exposure to cadmium through inhalation is considered to be a trigger for the activation of macrophages in a pro-inflammatory state. It is suggested that this initiates and liberates a specific form of RA through the formation of pulmonary nodules [133,146]. Significantly increased cadmium concentrations were observed, among others, in smokers suffering from RA [140]. There is evidence to suggest a link between the development of moderate and severe forms of osteoarthritis and the use of tobacco and the associated higher Cd concentration in the blood serum. Cd may trigger oxidative stress and inflammation, promoting cartilage loss [147]. Research conducted by Shiue [148] suggested a relationship between high antimony, tungsten, cadmium, uranium, and trimethylarsine oxide concentrations in the urine and the severity of pathological changes in the cervical spine of patients with AS. Abnormal levels of these elements and compounds can induce oxidative stress in patients with AS. Cadmium exposure was also proven to be a factor in induction and/or exacerbation mechanisms in the development of autoimmune joint diseases. These mechanisms include oxidative stress, disruptions in biogenic element metabolisms such as zinc and iron, and inflammation [149].

Vanadium also affects the NF-κB signaling pathway, leading to its activation [150]. The above studies confirm the involvement of vanadium in the potential pathogenesis of autoimmune processes underlying diseases such as RA, psoriatic arthritis (PsA), and AS [151].

**Figure 3:**
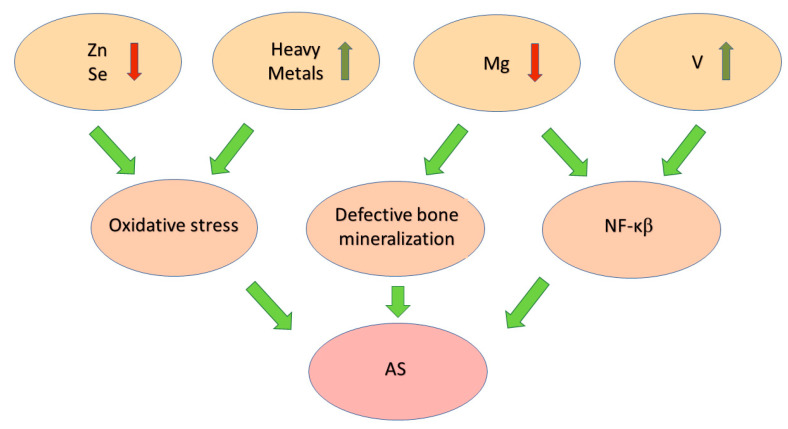
The schematic shows abnormalities in element concentration and their influence on the pathomechanisms underlying AS.

A summary of elements’ influence on the pathomechanisms of AS is shown in the schematic below [Figure 3].

Destabilization in bone mineralization processes in the body results in remodeling of the extracellular matrix, inflammation, and calcification. Hence, calcium, magnesium, and phosphorus levels play significant roles in the pathogenesis of AS. In turn, selenium and zinc are closely related to the functioning of the antioxidant barrier, and, therefore, the neutralization of free radicals, which accumulate in the described disease.

## 4. Lifestyle as an Environmental Factor in AS Development

Smoking is one of most common addictions related to pathogenesis of diseases. In addition to contributing to the pathophysiology of a number of rheumatic diseases, smoking increases the difficulty of therapy for patients when compared to non-smoking patients [152,153,154]. According to certain research, smoking can cause rheumatoid arthritis, which carries a higher risk of functional disability due to elevated risk factors, more severe disease activity, and considerable arthrosis damage [155,156]. Cigarette smoking has been linked to the advancement of spinal radiography in patients with early axial spondyloarthritis, according to Poddubnyy et al. [7]. An epidemiological study with 75 AS patients in Taiwan also revealed that smoking AS patients had significantly worse physical mobility parameters than non-smoking AS patients, including modified Schober’s index, cervical rotation, later lumbar flexion, chest expansion, and occiput-to-wall distances. Smoking AS patients also had significantly higher systemic inflammation parameters, such as ESR [157]. The study performed by Zhang et al. confirmed that smoking negatively impacts the physical functioning and disease activity of patients with AS [158]. Zhao et al. [159], in a large cross-sectional study, showed unequivocal evidence that both past and current smoking are associated with incrementally worse disease across a wide range of severity measures using a large and well-characterized national cohort of patients with axial spondyloarthropathies. Moreover, it was found that current smoking was associated with lower odds of acute anterior uveitis (AAU) and higher odds of psoriasis than either ex- or never smokers. A recent study showed that smoking can influence the development of AS, leading to particular pathological changes in the hip joints. Exposure to fewer than 10 pack-years of smoke can also increase the prevalence of hip involvement in AS [160]. The summary of smoking influence on AS patients is shown in Table 2.

The association between alcohol consumption and AS is currently underreported. A 1998 study based on national sickness insurance data in Finland established that excessive alcohol consumption was a significant factor contributing to the excess number of accidents and violent deaths among Finnish patients with AS. The average lifespan of patients with AS was 6–8 years shorter than the average lifespan of the Finnish population [161]. Zhang et al. reported no statistically significant variation in the analysis of the relationship between alcohol consumption and disease activity [158]. According to Zhao et al.’s findings, drinking alcohol is linked to lower measurements of the severity of axSpA disease compared to abstainers, although the author suggested a wider study on that matter due to the limitations of their study [162]. Research conducted by Min et al. found a substantial correlation between alcohol consumption and the advancement of spinal structural damage in individuals with axial spondyloarthropathies (axSpA). These findings suggest that alcohol consumption may have a deleterious impact on the development of spinal structural damage in individuals with axSpA [163].

Du et al. conducted research regarding the influence of work exposure on ankylosing spondylitis. The study compared the influence of three types of work: working in shifts, heavy lifting work, and mainly standing or walking work. It showed that there is no direct link between AS and the type of work exposure [164]. On the other hand, there are studies showing a link between the disease and physical activity. The two-sample Mendelian randomization proved the casual association between AS and the duration of physical exercise, which has been shown to be protective against its risk [165].

The other important factor that may affect AS patients is the quality of their sleep. Batmaz et al. proved that patients with AS suffer from pain, depression, and a low quality of life, which are correlated with a poor sleep quality [166]. The research conducted by Yüce et al. proved that poor sleep, depressive symptoms, and a poor quality of life might have a negative impact on the diseases [167]. Therefore, sleep quality analysis may be a good indicator to evaluate AS patients [168].

In patients with AS, worse disease outcomes have been linked to long-term exposure to air pollution and a high-fat diet [103]. Diet as an important factor in AS was also proven by Vergne-Salle et al. The study confirmed that an insufficient consumption of omega-3 polyunsaturated fatty acids and fiber was linked to elevated spondyloarthritis (SpA) activity. Additionally, a higher intake of ultra-transformed foods was found to be associated with increased SpA activity in a more comprehensive analysis. These findings support the idea that omega-3 polyunsaturated fatty acids and fiber play a role in decreasing immuno-inflammatory responses and preserving the integrity of the intestinal barrier. However, additional research is needed to fully understand the underlying mechanism of ultra-transformed foods [169]. The researchers Kao et al. stated that exposure to ambient CO can be an aggravating factor, but exposure to ambient NO_2_ can be a protective factor regarding the use of biologics indicated for high AS activity [170].

Research shows that lifestyle has a significant impact on the development of a given disease. For direct health behaviors, lifestyle factors include low physical activity, smoking cigarettes, drinking large amounts of alcohol, or a diet rich in highly processed fats and sugars. Appropriate lifestyle modification is necessary to minimize the risk occurrence of lifestyle diseases, including AS.

## 5. Infection in AS

Numerous studies have explored AS-related infections, including bacterial [171,172,173,174], viral [172,175,176,177], fungal [178], and microorganisms with sizes between bacteria and viruses [173,179,180]. Infected sites include the respiratory [173,179,180,181], immunological [176,177,179,180,181,182], digestive [171,173,177,179,180,181,182,183], and genitourinary systems [178]. However, there is no unanimity in the link between infections and the risk of AS.

Case–control and cohort investigations have proven that infections are found to increase the risk of AS. These findings are consistent across the majority of investigations, including four Asian studies [174,175,179,182], two European studies [172,183], and one North American study [176]. However, Bartels et al. found the opposite [171], indicating that past *Helicobacter pylori* (*H. pylori*) infection may lessen the likelihood of acquiring this disease. Another study discovered that H. pylori infection was eradicated in more than 80% of cases in the same cohort as Bartels et al. [184], implying that, when H. pylori is eradicated, it leaves a protective potential for the development of AS later in life [171]. Furthermore, the microbiota in the gastrointestinal tract change during H. pylori eradication, which may influence AS development [185,186]. Regarding infection types, we found no evidence that bacterial infections increase the incidence of AS. Only Keller et al.’s case–control study found a relationship between AS and a prior diagnosis of chronic periodontitis, which is defined by an oral bacterial infection [174]. This could be because rheumatic illnesses and chronic periodontitis have similar pathogenic features, such as inflammatory mechanism malfunction and an imbalance of proinflammatory and anti-inflammatory cytokines [174,187,188,189]. In cohort studies, Bartels et al. found that H. pylori may be a protective factor for AS [171]. Nielsen et al. found that bacterial infections are linked to the development of AS in the general population [172]. AS caused by different forms of infection is not well understood. For example, one study proposed that exposure to Candida albicans could induce AS via a T-cell-driven model of Th17 responses [175]. Another study found that Mycoplasma pneumoniae had a considerable impact on immune cells and the host’s immune system, including the polyclonal stimulation of T and B cells and the release of associated cytokines [179], resulting in a breakdown in immunological tolerance. In addition, subgroup analysis revealed that viruses play a significant role in the risk of AS in cohort studies. According to one study, viruses (such as human papillomavirus) may cause inflammatory or immune-mediated disease by activating the pathogenic IL-23/IL-17 axis, which results in higher serum levels of Th17 cells, IL-17, and IL-23, as well as an imbalance of IL-17A/IL-23 cytokines [178]. In a subgroup analysis of infection sites, we discovered that immune system infections were strongly related to the incidence of AS in case–control studies. Some immunological organs, such as the tonsils, play a role in allergen tolerance by producing allergen-specific FOXP3+ regulatory T cells, implying that they are essential in the establishment of immune tolerance [190]. Some investigations have hypothesized that the modification of immunological tolerance in tonsillitis patients can lead to inflammatory problems in autoimmune arthritis, including AS; hence, tonsillitis could be exacerbated by spondylitis, leading to the diagnosis of AS [181,182]. Furthermore, the increased incidence of AS among infected individuals may be explained by HIV-induced antigen-driven immune responses [191], T cell imbalance [192], and molecular mimicry between HIV proteins and self-antigens [193]. In cohort studies, infections at other sites were strongly related to the incidence of AS, indicating that respiratory tract and genitourinary system infections may be the cause. The pathophysiology of AS caused by genitourinary infection is complex. In one study, it was suggested that human papillomavirus in the genitourinary system may cause AS by activating the IL-23/IL-17 axis [178]. Klebsiella pneumoniae in the respiratory system may cause a decrease in the number of particular T cells, indicating an insufficient host defense against Klebsiella, allowing AS to be impacted by bacterial antigens that reach the joint [173].

In conclusion, this article demonstrates that infections are associated with an elevated risk of AS, despite the substantial heterogeneity of the included studies. While the mechanism of infection and the effect of bacterial and viral infections on this disease have yet to be determined, more studies, particularly higher-quality prospective cohort studies and case–control studies, are needed to confirm a true cause-and-effect relationship between infections and the risk of developing AS.

The available data indicate that, the HLA system, and more specifically its subtypes involved in the pathogenesis of this disease unit, have a large share in AS. CD8+ T lymphocytes are presented with arthretogenic peptides from intestinal microbes in order to induce an immune response. Proteins with an abnormal folding structure then accumulate and increase the production of pro-inflammatory cytokines. While the etiopathogenesis of this unit is not fully known, it indicates a large proportion of infectious agents.

## 6. Microbiota in AS

Recent research has indicated that the presence and progression of AS are connected to alterations in the variety and composition of the gut microbiome. Zhou et al. [194] discovered, using metagenomic shotgun sequencing, that *Bacteroides coprophilus*, *Parabacteroides distasonis*, *Eubacterium siraeum*, *Acidaminococcus fermentans*, and *Prevotella copri* were more abundant in AS. On the other hand, *Enterococcus faecium* E980 and TX0133a01 were shown to be less abundant. In their study, Liu et al. [195] conducted 16S rRNA gene sequencing on stool samples obtained from both AS patients and healthy controls (HCs). They discovered that the relative abundance of *Bacteroidetes* was lower in AS cases compared to HCs. Conversely, the levels of *Firmicutes* and *Verrucobacterium* were higher in AS cases. In addition, particular gut microorganisms were linked to the disease activity of patients with this disease. Costello et al. [196] discovered higher levels of *Lachnospiriaceae*, *Ruminococcus*, *Rikenellaceae*, *Porphyromonadaceae*, and *Bacteroidaceae* but lower levels of *Veillonellaceae* and *Prevotellaceae* in the terminal ileum of patients with AS. These findings indicate that certain species rich in AS may potentially initiate autoimmunity. The ratio of *Firmicutes* to *Bacteroidetes* (F/B ratio) is commonly linked to the maintenance of a normal intestinal balance, and an elevated presence of certain species of Firmicutes leads to AS [195,197]. Furthermore, the researchers also noted alterations in metabolites both within and outside the gastrointestinal tract in patients with AS. Alterations in intestinal metabolites are intricately linked to the metabolism of intestinal microbes, whereas modifications in extra-intestinal metabolites may also be somewhat associated with the transmission of intestinal microbial metabolites. Several studies have also demonstrated the crucial functions of certain bacteria in the development of AS. The prevalence of Intestinal *Klebsiella* was strongly associated with disease activity in AS [198]. There is a strong correlation between *Klebsiella* antibodies and intestinal inflammation in patients with axial AS [199]. The discovery by Wen et al. showed that *Actinobacteria* are more abundant in patients with AS and may have a role in regulating the ubiquitination of IkB-a. Consequently, this triggers NF-kB signaling and facilitates the accumulation of proinflammatory cytokines in patients with AS [200]. Furthermore, the contact between fungi and bacteria, known as cross-kingdom interactions, may have a role in the advancement of AS. SpA has shown the presence of heightened antibodies against mannan, a component of the fungal cell wall [197].

Genetic variations that affect the functioning of genes, including CARD9 and interleukin (IL)23R, have been discovered. These genes have a role in controlling the body’s natural immunological response to fungi [201]. Multiple studies support the notion that the gut microbiota can heighten the likelihood of AS by interacting with HLA-B27. HLA-B27 transgenic rats exhibited SpA-like disease without any external intervention. This disease is dependent on the gut microbiota triggering the IL-23/IL-17 pathway in the inflamed colon and joints [202,203]. Curiously, rats that were genetically modified to have the HLA-B27 gene and grown in a sterile environment did not show any obvious signs of inflammation in their intestines and joints [204]. Conversely, the presence of typical luminal bacteria consistently and uniformly led to persistent inflammation in the colon, stomach, and joints of B27 transgenic rats [205]. This indicates that the participation of bacterial flora is essential for the development of HLA-B27-related diseases.

The IL-23/IL-17 axis plays a crucial role in the development of AS by affecting the immune system. IL-23 primarily induces the secretion of DCs and macrophages, which, in turn, stabilizes the characteristics of T helper 17 (Th17) cells. It also plays a crucial role in the differentiation of different subsets of cells that secrete IL-17 [206,207]. The Th17 cell was formerly believed to be the main producer of IL-17. Recent research has discovered that various types of lymphocytes, including CD8+ cytotoxic T cells, Tc17 cells, γδ T cells, MAIT cells, NK cells, and ILCs cells, are capable of producing significant amounts of IL-17 [208,209,210]. IL-17 enhances the activation of T cells and induces the secretion of pro-inflammatory cytokines and chemokines by fibroblasts, epithelial cells, endothelial cells, and immune cells such as macrophages [211].

Intestinal inflammation in HLA-B27/β2 microglobulin (β2m) transgenic rats is accompanied by an elevated production of IL-23 and IL-17 in the colon tissue [212]. The interaction between cell surface ligands and particular bacteria triggers the development of cells that secrete IL-17 and IL-22, therefore determining their ability to cause disease [213,214]. Dysbiosis, which is the imbalance of microbial communities, can lead to an overabundance of Prevotella species at mucosal sites. This can have a direct or indirect impact on type 17 immune responses by changing the levels of microbial metabolites or affecting barrier function. As a result, immune tolerance can be disrupted and there can be an increase in pro-inflammatory cytokines like IL-23, which can trigger ankylosing spondylitis in susceptible populations. Prevotella primarily stimulates TLR2, leading to the production of Th17-polarizing cytokines, such as IL-1 and IL-23, by antigen-presenting cells. Prevotella further induces the secretion of IL-6, IL-8, and CCL20 by epithelial cells, thus facilitating the recruitment of neutrophils to the mucosal area. This mechanism results in the widespread spread of bacteria, bacterial metabolites, and inflammatory mediators, which, in turn, affects the outcomes of systemic diseases [215]. These findings indicate that an imbalance of microorganisms in the body leads to alterations in the balance of the immune system in both the intestines and joints, resulting in inflammation. This process is mediated by the IL-23/IL-17 pathway in AS. The bacterial diversity of the gut microbiota affects the balance between Th17 and Treg cells in the lamina propria (LP), which, in turn, can impact gut immunity, tolerance, and susceptibility to AS. Segmented filamentous bacteria (SFB) stimulate the synthesis of serum amyloid A (SAA) in epithelial cells, which, in turn, triggers the production of interleukin-6 (IL-6) and interleukin-23 (IL-23) by dendritic cells (DCs). This ultimately leads to the development of Th17 cells [216]. SFB induces aberrant T cell differentiation in the mouse gut via TLR5 [217]. The differentiation of Th17 cells is also associated with the presence of *Cytophaga-Flavobacterium-Bacteroides* (CFB) bacteria in the gut. This process is not influenced by Toll-like receptors (TLRs), interleukin-21 (IL-21), or interleukin-23 (IL-23) signaling, but it does depend on the appropriate activation of transforming growth factor beta (TGF-β). The absence of Th17 cell stimulation by bacteria is associated with an elevation in Foxp3+ Treg in the lamina propria [218]. Tregs were predominantly found in the colonic mucosa of mice. The guts of AS patients exhibited a prevalence of active Treg responses characterized by the production of IL-10. Additionally, there was a significant decrease in the number of Treg cells in the lamina propria of germ-free mice. However, the lost bacteria can potentially be restored with the addition of specific bacteria, such as *Bacteroides fragilis*, *Clostridium consortium* (particularly Cluster IV and XIVa), and the “altered Scheidler flora” (a combination of eight recognized symbiotic bacteria) [219,220,221]. Polysaccharide A (PSA), the chemical in *B. fragilis* which affects the immune system, stimulates the conversion of CD4+ T cells into Foxp3 + Treg cells. These cells play an active role in maintaining tolerance in the mucosal lining by generating IL-10 in the presence of normal bacteria [222]. It may be deduced that the immune system’s cells detect and are influenced by the metabolic byproducts, which, in turn, impact the equilibrium between pro-inflammatory and anti-inflammatory cells. For example, it has been documented that *C. Consortium* and *B. Fragilis* can stimulate the development of Treg cells by generating short-chain fatty acids (SCFAs) from carbohydrates found in the diet [223]. Recent data suggest that gut microorganisms play a role in the development of AS. The gut microbiome undergoes continuous and fluctuating changes. Hence, specific microorganisms can serve as markers for monitoring disease activity and assessing the efficacy of treatment. To accurately diagnose and treat patients with AS in the future, clinicians may need to perform rapid and thorough analyses of the gut flora [224].

Therefore, there are many studies that indicate that a relationship between the composition of the microbiome and the development of autoimmune diseases, including AS. The bacteria that are found in the intestines can act in two ways: to silence or intensify the immune response. This way, modified cells can migrate from the intestines to the joints, contributing, for example, to their reactive inflammation. From this point of view, lifestyle factors, in particular, a proper diet and the appropriate contribution of micro- and macro-elements, are important.

## 7. Genetic Polymorphism in HLA System

With 231 protein subtypes, the HLA-B27 family exhibits a high degree of genetic polymorphism. These subtypes differ from one another by only a small number of amino acids, which may change the molecule’s ability to bind peptides [3,225].

Human MHC, called the HLA complex, belongs to cell surface proteins operating in the process of acquired immunity. There are three subgroups in the MHC gene family: class I, II, and III. MHC class I encodes HLA-A, HLA-B, and HLA-C and is present on all human nucleated cells and platelets, presenting epitopes to T cell receptors (TCR) on the surface of cytotoxic T lymphocytes [3]. The heterodimeric subgroup of MHC class I consists of a polymorphic heavy chain. The chain contains three domains, i.e., α1, α2, and α3. The α1 domain binds non-covalently to the non-MHC β2m molecule, while α3 spans the cell membrane and interacts with the T cell CD8 coreceptor [226,227,228]. The MHC class I complex can connect to peptides 8–10 amino acids long through one cleavage separated from both α1 and α2, which leads to the initiation and propagation of the immune response [229]. A stable MHC molecule must be properly packaged and then folded in the endoplasmic reticulum of cell organelles (ER) under the guidance of chaperone proteins (calreticulin and tapasin) [226]. Although the classical MHC I class contains one heavy chain, there are three distinct MHC-I structures, including cell surface HLA-B27 homodimers and intracellular and exosomal MHC-I dimers [230]. These components may function in various pathophysiological processes. The exact mechanism binding the HLA-B27 antigen and AS has not yet been identified, but it is assumed that the intracellular process of HLA-B27 antigen formation is the subject of further research in this area [231]. Studies have shown that 85% of AS patients are characterized by the presence of the classic HLA-B27 allele. This does not mean, however, that this allele is responsible for causing the disease, because only 1–5% of carriers develop AS [232,233]. The arthritis-causing peptide hypothesis assumes that structurally mutually exclusive peptide–MHC complexes can directly initiate the specific HLA-B27 autoimmune response, relying on the primary structure of antigenic peptides [234]. Some microbial peptides are similar to self-peptides in body tissues and can activate the response of some HLA-B27-specific CD8+ T cells. T cells react with HLA-B27 peptide complexes, leading to autoreactivity and autoimmune disease [235].

HLA-B27 tends to misfold within the endoplasmic reticulum, leading to the formation of dimers and multimeters. Without the proper folding, HLA-B27 would be produced and transported to the cell surface as homodimers containing only heavy chains. The presence of such HLA-B27 variants correlates with the occurrence of AS [236]. Such misfolded proteins accumulate in the endoplasmic reticulum, activating autophagy and the IL-23/IL-17 pathway. They can also affect the functioning of the endoplasmic reticulum, causing a pro-inflammatory response to the presence of misfolded proteins, which, in turn, leads to the activation of the above-mentioned pathway [237]. Another hypothesis suggests that HLA-B27 homodimers are associated with the receptors on NK cells, myelomonocytes and lymphocytes. Binding occurs through the killer cell immunoglobulin-like receptor (KIR) and the leukocyte immunoglobulin-like receptor (LILR). This leads to the release of pro-inflammatory cytokines such as IL-17, TNF-α, and IFN-γ [238]. Although attempts have been made to demonstrate associations between HLAs other than B-27, particularly with HLA-B40 [239,240,241,242] and HLA-A02 [243], most of them have not been confirmed or repeated in independent studies. The only confirmed case of an antigen that may be important for the pathomechanism of AS is HLA-B14. The reports do not explain how this antigen contributes to the development of AS, but confirm its occurrence in a population of patients in whom HLA-B27 has not been detected [244,245].

Some researchers look for connections between AS and other genetic polymorphisms. In the study by Remans et al. [246], it was found that the CTLA4 gene has a protective effect against the phenomenon of oxidative stress, which may suggest that the presence of the discussed polymorphism actually reduces the antioxidant potential, but there are no reports in the literature that the polymorphism of the discussed gene has been tested in this aspect. To confirm the conclusions described, studies should be carried out on a larger population of patients with the described polymorphism. The research conducted by Dahmani et al. established a link between ankylosing spondylitis and the CT60/rs3087243 polymorphism of the CTLA4 gene. The study found that the HLA-B27 antigen and variations in the CTLA4 3’UTR region played an essential role in ankylosing spondylitis susceptibility in a west Algerian population. The genetic difference observed between the B27+ and B27− groups explains the disease’s variability [247]. STAT4 gene polymorphism is also important for the functioning of antioxidant enzymes in the bodies of patients with RA, PsA, and AS. In research conducted by Liu et al. [248] and Ebrahimiyan et al. [249], the involvement of the T allele of the STAT4 gene polymorphism in the occurrence of rheumatoid diseases such as RA and AS was confirmed. Research by García-Ruiz et al. [250] stated, however, that it is the high level of MDA that stimulates the expression of the COL1A1 gene. However, there is a lack of research in the literature presenting the above phenomena on a broader scale.

In recent years, researchers’ interest has focused on attempts to study genes outside the major histocompatibility complex. This seems important, because the etiopathogenesis of this disease is still not fully understood; therefore, it seems that the relationship between genes and AS remains one of the strongest and can be used to determine susceptibility to AS. Examples of such genes have been collected and presented in the Table 3.

## 8. Conclusions

Comprehensive medical care for a patient with AS should also include a detailed analysis of lifestyle and factors that may increase oxidative stress, such as smoking or excessive alcohol consumption.

The review carried out in this study indicates the involvement of many factors underlying AS. Factors related to lifestyle should be mentioned here, in which smoking cigarettes comes to the fore and, consequently, the production of free radicals. This is important, because the disruption of the pro- and antioxidant balance triggers a cascade of reactions that produce toxic products. In turn, the inflammatory process that accompanies this disease results in the release of a number of mediators and molecules that change the functions performed by cells, leading to the activation of pathological reactions. Moreover, proteins are secreted, the presence of which indicates an ongoing chronic inflammatory process. Another factor that plays a role in the pathomechanism of AS is exposure to heavy metals and environmental pollutants. The role of genetic factors is also important. There is a relationship between the occurrence of specific polymorphisms and, for example, a reduction in antioxidant potential, which may lead to the development of a condition called oxidative stress, which is related to the pathomechanism of AS. The data contained in this review constitute valuable information and encourage the initiation and development of research in this area.

## Figures and Tables

**Table 1:** The changes in oxidative stress parameters in AS patients in comparison to healthy controls.

Parameter	Result in AS Patients	Reference
SOD	Increased	[76,78]
	Decreased	[76,97]
	No significant change	[67]
CAT	Increased	[76,78]
	Decreased	[76,97]
GPx	Increased	[76]
	Decreased	[76,97]
GST	Decreased	[76]
GR	Increased	[98]
	Decreased	[76]
TAS	Decreased	[45,76,79]
TOS	Increased	[45,98]
MDA	Increased	[34,67,97,98]
AOPP	Increased	[97]
CP	Increased	[80,81,82]

**Table 2:** The influence of smoking in AS patients.

Factor	Participation in AS	Population	References
Smoking	Decreased physical mobility parameters including modified Schober’s index, cervical rotation, later lumbar flexion, chest expansion, and occiput-to-wall distances. Increased systemic inflammation parameters	Chinese	[157]
	Exposure of no more than ten pack-years may contribute to a higher prevalence of hip involvement in AS	Chinese	[160]
	Increased risk of psoriasis, decreased risk of acute anterior uveitis	United Kingdom	[159]
	Smoking is associated with spinal radiographic progression in patients in first 10 years of the disease	German	[7]

**Table 3:** New genetic polymorphisms’ influences on AS.

Polymorphism	Genotype	Influence on AS	Population	References
IL-1A-889 (*rs1800587*)	various	increases the risk of AS in studied populations	English and Tunisian	[251]
IL1F7 exon 2 (*rs3811047*)	G allele	is negatively correlated with susceptibility to AS	Canadian and Chinese	[251]
IL5 (*rs2069812*)	A allele	associated with lower CRP and VAS values	Polish	[252]
IL9 (*rs2069885*)	A allele	associated with lower CRP and VAS values	Polish	[252]
IL17F (*rs763780*)	G allele	should be considered as a promising biomarker of disease activity and anti-TNF treatment outcome	Polish	[253]
IL17RA (*rs48419554*)	G allele	may serve as a potential marker of disease severity in Polish AS patients	Polish	[253]
TLR2 (*rs5743708*)	A allele	haplotypes appear to be involved in the development of clinical forms of SpA and can be a possible therapeutic target for the spondyloarthritis.	Brasil	[254]
TLR9 (*rs187084_rs5743836*)	T/C allele	haplotypes appear to be involved in the development of clinical forms of SpA and can be a possible therapeutic target for the spondyloarthritis.	Brasil	[254]
TIMP3 (*rs11547635*)		may be associated with susceptibility to AS	Chinese	[255]
RUNX3 (*rs760805*)	T allele	can contribute to AS incidence	Chinese	[256]
TNFα (*rs1799724*)	C vs T allele	reduced risk of AS	Asians	[257]
TNFα (*rs1800629*)	G vs A allele	significantly increased the risk of AS in Caucasians and decreased the risk of AS in mixed populations.	Asians, Caucasian	[257]
TNFα (*rs361525*)	G vs A allele, GG vs GA genotype	linked to an elevated AS susceptibility	Asians	[257]
TNFα (*rs1800630*)	C allele	linked to an elevated AS susceptibility	Asians	[257]
TNFAIP3 (*rs10499194*)	T allele, CT genotype	may be associated with a reduced risk of AS.	Chinese Han	[258]

## Data Availability

Not applicable.
